# A Rapid Fluorescence Method for In Vivo Quantitation of Lung Deposition of a Nebulized Drug: Multiple Uses for Advancing Aerosolized Drug Development and Specific Insight Regarding Aerosolized Vitamin A for Preventing Bronchopulmonary Dysplasia

**DOI:** 10.3390/mps8060140

**Published:** 2025-11-14

**Authors:** Craig A. Gelfand, Ying Wang, Gourav Chandan, Jie Liu, Sabrina Madrigal, Reiko Sakurai, Celia Yu, Catalina Guerra, Robert Segal, Virender K. Rehan

**Affiliations:** 1 Advent Therapeutics, 3805 Old Easton Road, Doylestown, PA 18902, USA; 2 Department of Pediatrics, The Lundquist Institute for Biomedical Innovation at Harbor-University of California, Los Angeles (UCLA) Medical Center, David Geffen School of Medicine at UCLA, 1124 W Carson Street, Torrance, CA 90502, USA; 3 The Lundquist Institute for Biomedical Innovation at Harbor-University of California, Los Angeles (UCLA) Medical Center, David Geffen School of Medicine at UCLA, 1124 W Carson Street, Torrance, CA 90502, USA

**Keywords:** inhalation dosing, aerosol dosing, fluorescence quantitation, in vivo dose quantitation, vitamin A, bronchopulmonary dysplasia

## Abstract

We have developed a method for in vivo quantitation of lung delivery of inhaled nebulized drugs by measuring a fluorescent-labeled analog in bronchioalveolar lavage fluid (BALF) collected immediately after inhalation dosing. The effectiveness of delivery of an aerosolized formulation of our proprietary water-miscible vitamin A product to the deep lung (target organ) was studied; the product is being developed for prevention of bronchopulmonary dysplasia (BPD) in preterm infants. The fluorescent retinol analog was incorporated by spiking into a standard formulation, remaining fully compatible with existing nebulizer administration procedures for animal exposure. The method provides quantitation of the delivered dose (DD) to the lung within a few minutes after dosing; fluorescence in BAL in a plate reader allows for simple rapid quantitation of the delivered drug, while avoiding the complexities of other labeling methods (e.g., heavy labels or radioactivity). Data from newborn rat and lamb models showed linear dose responses, validating the method. Approximately 5–10% of the inhaled drug was recovered in BALF in both models, consistent with reports in the literature. The ease of use of the method facilitated various aspects of our project, including the transition to more clinically relevant animal models and aerosol exposure systems. The formulation of this approach could be spiked into other formulations, allowing application of the method to other aerosol drug development programs.

## 1. Introduction

Inhaled delivered dose (DD) quantitation is an important metric at several stages of drug development. For example, for our continued efforts developing nebulized vitamin A to prevent bronchopulmonary dysplasia (BPD) in preterm infants, measuring lung DD became critical for bridging from a convenient whole-body aerosol exposure (WBAE) system [1] to the more clinically relevant nose-only aerosol exposure (NOAE; see, e.g., [2,3]) system. DD quantitation is additionally complicated by the shallow, rapid breathing of neonates (rat pups and lambs) limiting inhalation efficiency [4], and attributes (including age) that are not well accounted for in mathematical models [5,6] of drug inhalation. Methods for precise inhaled DD quantitation, among many hurdles in respiratory drug development (as reviewed, e.g., [2,7]), require specialized equipment and operator skill, which are often not available in vivarium settings, and assay and related method development can be slow and expensive. While radiolabeling offers both quantitation and spatial discrimination, it brings hazards of radioactive aerosol use and containment and the high cost of radiolabeled materials.

We have developed a simple and rapid in vivo DD quantitation method by incorporating a fluorescent vitamin A analog into our aqueous vitamin A formulation, without altering nebulizer setup or aerosol administration. The method encompasses key attributes preferable for early-stage R&D projects, namely speed, ease, and low cost. An available and safe (non-radioactive) label is quantitated in broncho-alveolar lavage fluid (BALF) with a simple fluorescence plate reader, yielding data in near real time and enabling rapid experiment iterations. Here we have focused solely on measuring fluorescence in BALF, generating sufficient data for early-stage aerosol R&D efforts, and allowing the deferral of costly, time-consuming, and complex efforts to establish more precise quantitation methods in multiple sample types (e.g., for PK/PD studies, rates of permeation into lung tissue or blood, etc.) until the drug is proven to deserve such efforts. We present several use cases in which this dose quantitation method can provide useful data during the development of most new aqueous aerosol drugs. We show how the data generated give particular insight into our own development of inhaled vitamin A for targeted deep lung delivery for the prevention of BPD [1].

## 2. Materials and Methods

### 2.1. Materials

Our proprietary formulation is vitamin A palmitate (DSM-Firmenich, Kaiseraugst, Switzerland) solubilized at 50,000 U/mL in polysorbate 80 (HX-2^®^, NOF America, New York, NY, USA). The specific fluorescence preparation has 3% of the vitamin A replaced with LightOx™ 23 (CAS 2342579-18-2, Millipore Sigma, St. Louis, MO, USA), a fluorescent vitamin A analog, which happens to retain similar biological activities [8], without requiring change to our formulation method or resulting physicochemical properties. All experiments used the same vibrating mesh nebulizer (Aeroneb^®^, Aerogen, Galway, Ireland), which outputs aerosolized particles of approx. 3.7 microns, based on laser diffraction of the vapor of nebulized vitamin A formulation, in the ideal size range for achieving deep lung deposition [9].

### 2.2. Aerosol Dosing to Rat Pups

Sprague–Dawley rat pups were at least 6–7-days old to ensure reproducible BAL. WBAE followed our previously established methods [1]. Pups were dosed simultaneously in groups of up to 8, usually keeping litters together. The nebulizer was loaded with 25 or 50 International Units (U) vitamin A per gram body mass, diluting with sterile saline as necessary. Generally, we nebulized 1 mL total volume, which fully nebulized in about 4 min. NOAE was performed using inExpose™ with SoftRestraints™ (SCIREQ, Montreal, QC, Canada), following manufacturer guidelines. The total duration of aerosol exposure, both for WBAE and NOAE, was approximately 10 min, ensuring complete inhalation of the nebulized dose and residual inhalable vapor. Animals were then immediately euthanized prior to BAL, using 0.15 mL of Euthasol^®^ (sodium pentobarbital 390 mg + sodium phenytoin 50 mg/mL; Virbac, Fort Worth, TX, USA).

### 2.3. Dosing to Lambs

Aerosol: Individual newborn lambs (age 1–12 days) received aerosol dosing with a total of 500 to 22,500 IU vitamin A in 2 mL total volume, diluted with sterile saline, with the nebulizer attached directly to a tight-fitting mask (JorVet, Jorgensen Laboratories, Loveland, CO, USA); dosing consistently emptied the nebulizer within 7–10 min. Dose level order was randomized. The overall handling time (including exposure and lavage) was less than 30 min, with the benefit that any ongoing study/exposure conditions need be only briefly interrupted.

Liquid instillate: Separately, direct-to-lung liquid instillate dosing was accomplished terminally using a 10 Fr catheter inserted directly through a tracheostomy followed immediately by 10 bag breaths at 25/6 (PIP/PEEP) cm of H_2_O pressure; this was performed only with the final dose in these lambs. Liquid instillation provides greater certainty of the lung delivery of doses (e.g., [10]) and avoids ambiguities from the loss of inhaled material en route from nose to lung (e.g., deposition of inhaled particles in the nose or upper airway, exhalation without deposition, etc.).

### 2.4. Bronchoalveolar Lavage

Rats: The trachea was cannulated using a 24 g catheter, and lungs were lavaged three times with a cumulative volume of 0.6 mL sterile water, resulting in approximately 300 μL of BALF. Sterile water was used for BAL in anticipation of potentially low levels of fluorescence in the tiny lungs, avoiding chloride in isotonic saline which could quench fluorescence and reduce signal intensity; a precaution that resulting data ultimately showed was likely unnecessary.

Lambs: Lambs first underwent anesthesia induction with 3.5 to 4.5% isoflurane via a face mask, with maintenance at 1 to 3% isoflurane throughout the procedure. BAL was performed using an endotracheal tube (3.5 or 4.0 mm depending on animal size), with saline instilled in two aliquots of 15/15 mL (PND 1–3) or 25/25 mL (PND ≥ 4), respectively, via a 10-gauge catheter. Aspiration of the instilled fluid was facilitated by attaching the catheter to a suction machine set at negative 75 mmHg pressure, applying bilateral gentle chest percussion, and briefly using Trendelenburg positioning, collecting the fluid into a suction trap. The aspirated fluid from the two separately instilled aliquots was pooled, typically yielding 1–3 mL total volume. Terminal BAL following liquid instillation used the same process, but aspiration was performed using 50 mL syringes. We note that lung lavage with isotonic saline was performed gently given the repeated dose/BAL performed on the lamb, and with the expectation that larger lung volumes would yield correspondingly higher fluorescence signal, mitigating the above-mentioned concern related to chloride ions.

### 2.5. Fluorescence Assay

BALF was centrifuged (2000× *g*, 5 min for rat, 14,000× *g*, 10 min for lamb, at 4 °C), and fluorescence intensity read using a BioTek Synergy H1 microplate reader (Agilent, Santa Clara, CA, USA), in 96-well black microtiter plates, using 356 nm excitation and 496 nm emission, usually with triplicate reads of each BALF sample (in neighboring wells). For data presentation, the dots on the graphs represent the average of triplicate readings of the BALF sample from one rat or one dose/lavage interaction for lambs. All prepared microtiter plates were kept covered, in the dark at room temperature, until reading. We aimed to minimize the time before reading the BALF samples, generally within 10 min after centrifugation, to avoid any unknown alterations to the sample or its fluorescence, but prepared standard curves can safely dwell longer (see Appendix A.2). A freshly prepared standard curve was made by the serial dilution of the to-be-dosed formulation (diluting in water or saline to match the BAL medium), correlating known total vitamin A concentration to observed fluorescence, such that the apparent level of total vitamin A present in each BALF sample can be extrapolated; it is this calculated total vitamin A level that is presented in the data. In the case of using this fluorescence formulation as a spiked tracer into other drug formulations, this extrapolation would similarly yield the total amount of drug inhaled and delivered. We define the measurement as DD_BAL_, the apparent amount of DD recovered in the BALF, as the sample representative of the dose delivered to the lung. When needed, BALF was diluted to achieve fluorescence intensity in the responsive range of the standard curve, to ensure avoiding fluorescence inner filter effects.

### 2.6. In Vitro Measurement of ‘At the Nose’ DD in WBAE System

**Scheme 1:**
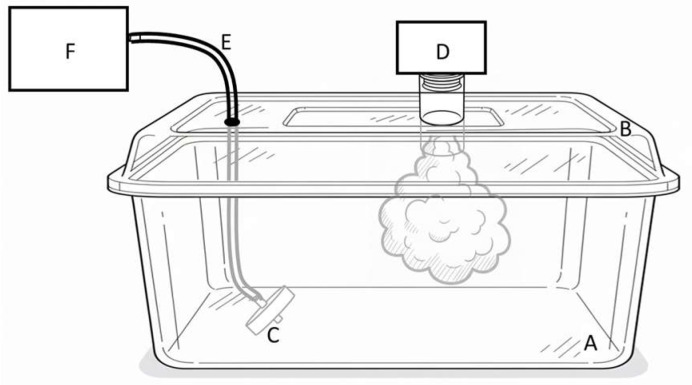
Schematic of in vitro WBAE DD_N_ measurement system, with components as follows. A = animal chamber. B = custom-built lid to hold nebulizer and tubing. C = membrane holder. D = nebulizer, mounted in place to nebulize downward into the chamber. E = connector tubing. F = digitally controlled syringe pump. Not drawn to scale. Note: The system configured for in vivo WBAE uses components A, B, and D, where B has only the orifice for mounting D (without through-hole needed for E).

These data are used as a comparator for the in vivo data above. The experimental setup (Scheme 1) is operationally similar to the DD calculation suggested by Bide et al. and Alexander et al., among others [5,6]. We used the same WBAE chamber used for in vivo exposure, a typical animal housing ‘cage’ (Ancare, Bellmore, New York, NY, USA), approx. 25 × 15 × 12.5 cm (approx. 4.7 L airspace volume), with a custom-built lid holding the nebulizer centered over the WBAE chamber; for these experiments a small hole in the lid allows positioning of the in vitro ‘nose’ part. The ‘nose’ is a Teflon membrane holder, the orifice of which is fortuitously about the same size as a rat pup nose, which is left unobstructed for ‘inhalation’ of vapor. The other end of the holder is connected with tubing to a syringe pump (NE-1000, New Era Pump Systems, Farmingdale, NY, USA) used to mimic the inhalation portion of tidal breathing. The tubing is held tightly in an exact-sized hole in the lid, such that the holder hangs with the orifice about 1 cm off the chamber floor, approximating the position of a rat pup’s nose.

Aerosolization simulated the setup for dosing to eight PD7 rats (approx. 11 g each), loading the nebulizer with 25 U/g, i.e., 2200 U total, diluted to a final volume of 1 mL; with this volume, the nebulizer runs to empty in about 4 min but the syringe pump was run for a total of 8 min to ensure ‘inhalation’ until the aerosolized mist had visually fully settled and/or condensed on surfaces (matching our standard in vivo WBAE protocol with dwell in the box for 6–8 min after the nebulizer ran to empty). The syringe pump was set to withdraw chamber air though the filter at a constant rate of 25 mL/min, a representative respiratory minute volume (RMV), with aerosolized particles being accumulated on the filter. RMV for S-D rat pups has been measured to range from approx. 12.5 mL/min at PD1 to 44 at PD10 [11], consistent with ranges predicted by commonly applied extrapolations based on body weight (bw) such as approx. 8.5–23 [6] or 17–37 mL/min [12] (noting that actual bw of the neonatal rats is below the range used to define the equations of [6,12]). Membranes were eluted with 20% ethanol/80% water, and eluent measured for OD325 nm, the absorbance maximum of vitamin A. Fluorescence could have been used with similar results, but in this case the extinction coefficient of vitamin A is sufficient to give an unambiguous signal (whereas absorbance by itself proved to be insufficient for any in vivo measurements—essentially leading us to develop this fluorescence method). Control experiments confirmed linear and quantitative recovery from the membrane of manually spotted vitamin A formulation, with an expected essentially zero background signal, and, separately, that quantitative capture of aerosol is achieved with a single membrane by showing that a second membrane, connected serially immediately behind the first membrane, had no detectable vitamin A captured.

## 3. Results

### 3.1. In Vivo Quantitation of Lung DD from WBAE in Newborn Rat Pups

**Figure 1:**
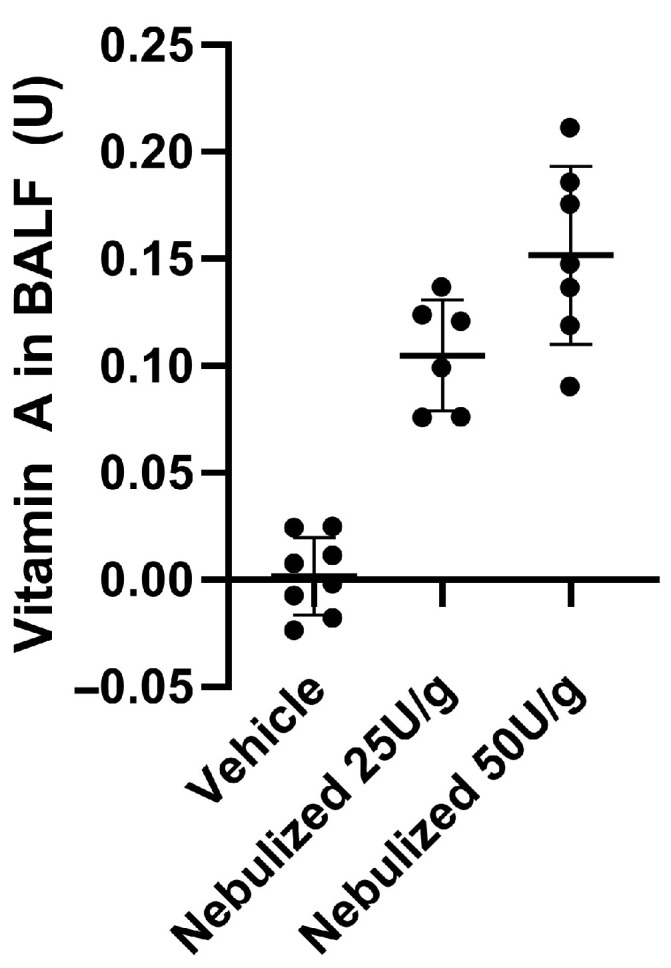
Quantitation of inhaled dose in WBAE dosing system. Healthy PD = 7 rats (4 m, 4 f in each group) were exposed to a single dose of the fluorescence-bearing vitamin A formulation or saline (nebulizer vehicle), with nebulizer loading as shown. Rats were sacrificed immediately after exposure, and BALF quickly collected and fluorescence intensity was read. The total amount of vitamin A (U) in BALF is reported, calculated in relation to a freshly prepared standard curve made from the same stock used for nebulization. *n* = 6–8 per group; average and standard deviation are shown. The vehicle-dosed group shows 0.0 ± 0.2 U, confirming the absence of a fluorescence background signal.

In vivo DD detected in BALF (DD_BAL_) was determined using single dosing administration, following standard dosing conditions described previously ([1,13,14,15]), and immediately performing BAL post mortem to ensure proper lung access, full and reproducible lung expansion, and sample collection adequacy. We compared vitamin A in BAL after exposure to aerosolization of the nebulizer *loaded with* 25 U/g or 50 U/g, and a vehicle-dosed litter as a control group (Figure 1); a clear dose–response relationship was observed. Data can also be expressed as DD_BAL_ per g of body mass: approximately 0.0091 U/g and 0.014 U/g.

### 3.2. Optimization of the NOAE Exposure System for Maximal Dose Delivery

NOAE optimization typically relies on assumptions (e.g., predicted tidal volume, density of nebulized mist, etc.), presenting uncertainties that can be avoided by obtaining quantitative in vivo DD data. Extending inhalation duration in pups produces hypothermia, presumably exacerbated by the convective cooling mechanism of the bias flow itself, and is thus not feasible. Bias flow needs to be optimized to maximize dose inhalation within 10 min, and within the same overall dose-plus-handling timeframe safely used for WBAE of approximately 20 min.

**Figure 2:**
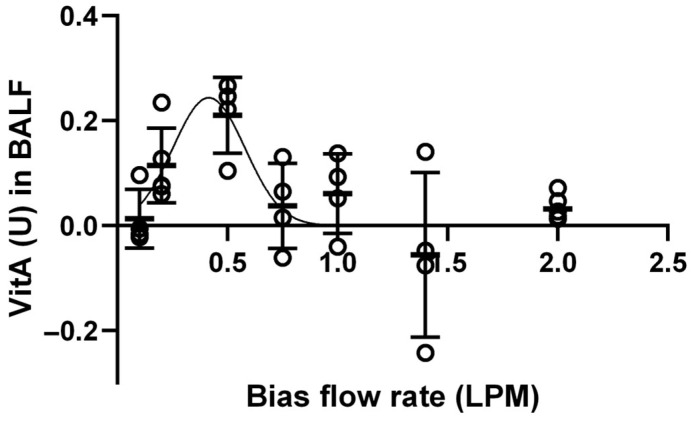
Amount of vitamin A in BALF of rat pups (PND = 7) as a function of bias flow setting of nose-only dosing. Data are *n* = 4 per bias flow, with average and standard deviation shown. Grubbs’ test was applied to each group, but no outliers were identified for removal. A Gaussian fit line (GraphPad Prism version 10.2.3 for Windows, GraphPad Software, Boston, MA USA) is shown.

The dependence of the BALF-recovered dose on bias flow was determined (Figure 2), with *n* = 4 PD7 rat pups exposed simultaneously per bias flow setting. A Gaussian fit to the data depicts an optimum around 0.43 LPM, but we viewed further experiments to not merit the additional consumption of animals for likely little gain, so we have defined 0.5 LP bias flow as our standard for future work. From these data, the apparent maximal dose is approx. 0.21 U, or 0.019 U/g. We noted single outliers in some groups, and for these we ruled out body mispositioning, as operators did not note deviation from proper nose positioning. Although further evaluation of the underlying cause for these outliers is out of scope for the current study, we hypothesize that transient alteration of respiration due to handling (the pups were not anesthetized) may be a plausible cause.

### 3.3. Deposited Dose Quantitation in Newborn Lamb

**Figure 3:**
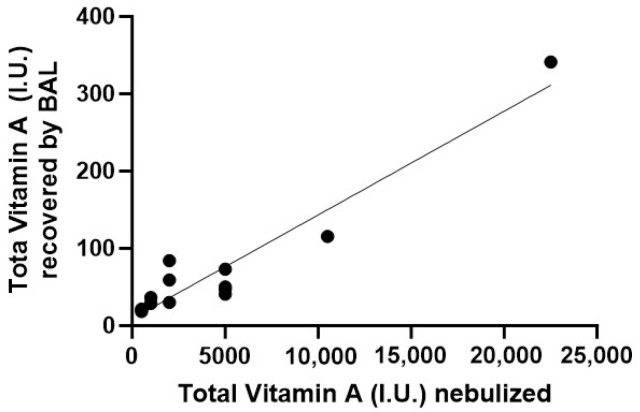
Vitamin A levels in BALF from lambs (PND = 1–15) recovered as a function of total amount of vitamin A loaded into nebulizer, while maintaining a total nebulizer load of 2 mL volume. Results are from 15 different dose events, performed in random order, accumulated from repeat interventions on 3 different lambs. The linear extrapolation gives r^2^ = 0.93 and intercept = 11, the latter recapitulating the very near zero fluorescence signal appropriate in the absence of dosing.

A range of dose levels were tested (Figure 3), leveraging the benefit that these larger animals offer safe and reliable repeat dose/BAL interventions (we allowed at least 24 h between experiments for lungs to normalize). The two highest doses yielded such high fluorescence that BALF had to be diluted to avoid inner filter effect (avoiding self-absorption of fluorescence emission; see, e.g., [16]). The fit line calculates recovery of about 78 U from a 5000 U nebulizer load, or DD_BAL_ of 0.014 U/g (within range of 0.011 to 0.020 U/g). In separate experiments, liquid instillate dosing of 5000 U led to an average recovery of 647 U recovered in BAL performed ex vivo, while maintaining an overall time of experiment as similar as possible to in vivo protocols.

### 3.4. In Vitro ‘At the Nose’ Dose Measurement

To complement interpretation of the in vivo WBAE data, we used an in vitro model of rat pup inhalation capturing ‘inhaled’ aerosol on a membrane. From extensive replicate measurements of nebulizing 25 U/g, 2.0 ± 0.2 U vitA was captured on a single membrane, or in other words, the DD_N_ was 2.0 ± 0.2 U vitA for a typical PD = 7 rat pup’s nose. Importantly, these findings would scale directly with the *actual* RMV: for example, if the RMV is halved (about 12.5 mL/min), DD_N_ would scale as about 1.0 ± 0.1 U. The dose captured on the membrane in comparison to the far higher dose loaded into the nebulizer is merely a reflection of the large dead volume of the WBAE enclosure.

## 4. Discussion

We developed this method to rapidly and accurately quantify the in vivo inhaled dose of aerosolized vitamin A, increase operational efficiency, and reduce animal use. The method is particularly well suited for comparisons, such as of the delivery of different concentrations of drug or under different conditions, some of which are highlighted as use cases below.

### 4.1. Operational Attributes and Benefits

No special reagents are required other than the specific fluorescent formulation itself. Costs of special chemical labeling (radioactivity or heavy isotopes) are avoided, as is the operational challenge of containing the radioactive mist and associated animal handling issues (e.g., how to clean/decontaminate animals of non-inhaled radioactive material adherent to hair, skin, and nose, etc.). Also avoided is the need for complex analytical equipment, e.g., liquid chromatography (LC) or LC/mass spectrometry, and associated specific operator skills/training and maintenance. Overall, the method, from dosing to lavage to quantitation, takes only about 20 min, which is particularly useful for experiments requiring multiple iterations, e.g., our optimization of NOAE bias flow (Figure 2), or goals such as evaluation/optimization of alternative nebulizers or effects of animal/aerosol interfaces. By comparison, typical chemical analytical methods would at best require hours of analysis per dose iteration.

The fluorescence formulation is stable and the standard curves were highly reproducible (see Appendix A.2). Regardless, we opted for daily standard curves, which were easily and quickly prepared, to ensure an exact match to the to-be-nebulized material and ensure data consistency. Fluorescence background noise from light scatter, a typical concern when measuring biological samples, is minimized from both the long Stokes shift of these vitA analogs, 140 nm in this case, and centrifugation of BALF, which proved adequate for eliminating cells/debris. The standard curve shows an apparent lower limit of quantitation approaching 0.01 U vitamin A per milliliter, which is as little as one five-thousandth of the concentration loaded into the nebulizer, with such levels being more than sufficient for our project and probably sufficient to quantitate the DD of other aqueous aerosolized medicines as well. For context, a sample at 0.01 U vitA/mL contains approximately 0.5 nM of the fluorophore, which is essentially on par with easily achievable sensitivity levels for analyte-specific assays (e.g., ELISA) or general methods like LC/MS. Attempts to quantitate in serum were disappointing, with benchtop centrifugation unable to eliminate the significant light scattering of this cell/biochemical-rich sample, including from control serum samples. Although we have successfully quantitated fluorescence in BALF in our models, it may be possible that certain BALF samples, e.g., in cases of extensive lung damage, could contain higher levels of cellular contamination or dramatic increase in protein content (e.g., mucins in a COPD model) that might pose challenges from light scattering similar to our experience with serum. Like with any assay, fit-for-purpose would need to be demonstrated for systems in which respiratory impairment or lung damage can be anticipated a priori (e.g., a disease leading to low inspiratory or tidal volume or deep lung congestion). Of benefit, we note that the extent of such interference is easily discovered, simply by screening for spurious fluorescence signal in ‘blank’ (non-fluorophore-exposed) BALF samples; Figure 1 and Figure 3 in particular show how we incorporated evaluation of background into our overall experiment design and analysis. If necessary, it would be relatively straightforward to introduce sample cleanup processes such as ultracentrifugation, low-molecular-weight cut-off membrane filtration, and/or extraction to overcome light scattering interferences.

The fluorescence is in essence a tracer, and the formulation should be easily miscible into other aqueous formulations intended for nebulization, and thus theoretically could be used to quantitate delivery of many aqueously borne inhaled drugs. Data indicate that fluorescence in BALF is viable with nebulizing a 1:1000 dilution of the stock, which should enable ‘spiking’ into virtually any to-be-nebulized aqueous formulation. The fluorescence is stable during experiment timeframes, and the solubilized vitamin A (including the fluorescence labeling) is unlikely to interfere/interact in any significant way with hydrophilic medicines (also see Appendix A.1 and Appendix A.2). We posit that the detection sensitivity may also be advantageous when aerosol dosing is to be performed on animals with impaired breathing, such as the low RMV of neonates as in our case or in other pulmonary conditions like COPD, asthma, etc. In all of these conditions, in vivo measurements are even more valuable, because body-weight-based RMV models have been built primarily with respect to pulmonary metrics of healthy animals. Future study with fluorescence microscopy, beyond the scope of our current development efforts, could prove to have potential for revealing the spatial deposition of the inhaled vapor.

### 4.2. Use Cases: Examples of Facilitating Progression of Our Inhaled Drug Development Program

A variety of uses for this quantitative fluorescence assay as discussed below, highlighting how the resulting data helps expand our understanding of our model systems and the inhaled dosing method itself, and potentially sheds light on the benefits of the inhaled dosing route.

**Use case: optimizing NOAE and bridging from WBAE.** Rapidly iterated experiments to optimize the bias flow setting of the NOAE device were performed. An initial ‘optimal’ bias flow was set at 0.5 LPM for yielding the highest DD_BAL_, approx. 0.21 U, within the 10 min handling window that proved to be well tolerated and for reliable handling of the PD7 pups. As expected, NOAE proved to be more efficient at delivering a higher dose vs. WBAE (0.21 vs. 0.15 U/animal, respectively), due at least in part to the lower dead volume of the NOAE apparatus. Poor fluorescence yields at higher bias flow (Figure 2) are consistent with aerosol being displaced from the device faster than it can be inhaled, likely coupled with the low respiratory minute volume (RMV) of these small pups. We also clearly observed sub-optimal, ‘too-low’ bias flow settings, revealing a maximum result near 0.5 LPM. The results obtained were based on the collection of raw data and were not reliant on assumptions or mathematical models of the dosing system or animal physiology, as has been described in the literature (e.g., [6,12] among others). The availability of quantitated DD helps us preserve the context of all data, minimizing the risk of discontinuity (e.g., of dose level to biological response) in our evolution from WBAE to the routine use of the NOAE system, with the latter more aligned with regulatory expectations of targeted (e.g., nose-only) delivery of aerosol doses as part of drug development programs.

**Use case: quantitating lung deposition efficiency in rat pups.** The conventional wisdom for inhaled drugs holds that 5–10% of a nose-delivered nebulized drug reaches the lung [17,18,19,20,21,22], but there are a range of physical (aerosol properties) and physiological variables that can cause deviation from the convention. For example, in our case, the newborn animals in our studies had bws below the ranges typically used to define allometric scaling equations, primarily based on adult animals (as in [6,12]), therefore defining an a priori concern regarding whether or not those models could be accurately applied to our studies. For the rat pup WBAE system, the fluorescence method allowed us to quantitate DD_BAL_ from inhaled dosing to PD7 rat pups (in vivo exposure; Figure 1) and, separately, measure DD at the nose (DD_N_) (in vitro setup). The single ‘assumption’ in this case is the RMV for rat pups; as described above, their bw changes significantly over the course of 7–10 days after birth. In our in vitro measurement of DD_N_, we simulated RMV = 25 mL/min, a reasonable fit to PD7 RMV (per [11]), and we found that indeed 5–10% of the dose that reached the nose was recoverable and quantitated in BALF. The fact that our quantitation matches the conventional wisdom in this case could be interpreted as the data retrospectively mitigating our concern regarding bw differences between our study and the models, but this outcome does not detract from the importance of having the quantitation available. Having the ability to quantitate DD_BAL_ can overcome risks that are otherwise present when systems being studied are known a priori to deviate from the data underlying the definition of the allometric models. For example, it seems equally plausible that studies of animals with lung disease (e.g., an induced COPD model) might have breathing parameters that deviate from those allometric models, which are built primarily upon measurements in healthy animals; as such the allosteric model could result in potentially misleading data, for example, regarding a new drug’s efficacy, in the absence of direct quantification of DD.

**Use case: demonstration of scaling up from small (rat) to large (lamb) animal models.** The fluorescence method allowed quick and efficient verification that the dosing method in the lambs functioned as expected on scale-up from rats, i.e., ~400× larger animals (~11 g rats at PD7 vs. lambs ranging from 4 to 8 kg during their first ~2 weeks of life) with associated larger RMV, and a different aerosol delivery interface (tight-fitting nose cone with nebulizer attached for lamb vs. rat WBAE or NOAE with larger dead volumes for rat), etc. Of note, even from the first DD_BAL_ measurements in lambs, we confirmed that we can achieve similar U/g in the lung, an important outcome that supports further evaluation effectiveness of dosing in these two very disparately sized species. Importantly, the fluorescence method allowed us to minimize the number of animals used, thereby addressing ethical considerations of unnecessary animal usage, while also reducing cost/handling hurdles for larger animals such ewes and lambs.

**Use case: quantifying BALF recovery efficiency of aerosol-delivered products in lambs.** Because BAL-based quantitation of inhaled dosing can be technique-dependent, with variability from instilled volume, recovery success, and distal (deep lung) penetration, we benchmarked recovery using a known liquid instillate dose. The delivered dose volume and concentration of the instillate are known, allowing accurate calculation of BAL recovery efficiency. We recovered 13% of the instilled dose (5000 U vitamin A introduced; avg. 647 U recovered) by ex vivo BAL, yielding a correction factor that can be applied to in vivo DD_BAL_ results. Recovery efficiency is likely less of a concern in rat pups, where post-mortem lavage allowed visual confirmation of full inflation of lungs and thus more complete lavage. All studies were performed in duplicate to confirm reproducibility.

**Use case: quantitating lung deposition efficiency in lambs.** Maintaining the same operational parameters (such as lavage volume and time after dose), in vivo BAL recovery efficiency should mirror that of the liquid instillate procedure discussed above. Comparison to DD_BAL_ after inhalation from the replicates, in which 5000 IU was loaded into the nebulizer (which is the same U as instillate dosing), gives a direct estimate of overall nebulizer-to-lung efficiency. Applying the 13% BAL recovery efficiency correction factor (as above), the average DD_BAL_ is calculated as 424 U in the lung from 5000 U loaded into the nebulizer, equating to an approximate 8.5% dose delivery efficiency (presenting lung delivery as a percent of nebulizer load). The findings are consistent with the low dead volume of the tight-fitting muzzle cone with the nebulizer directly attached, an interface approach more similar to that expected to be employed in the clinic with a typical nebulizer/facemask setup.

### 4.3. Limitations of the Method

In light of the several considerations described above, our method could be considered as a ‘semi-quantitative’ technique, but which is still more accurate than indirect calculated methods that rely on a series of assumptions based on species, weight, and RMV (as described above). Extant deficiencies need not preclude robust *comparative studies* simply by ensuring that the lavage is performed quickly after dosing, and that operation timeframes and BAL protocols are consistent across all dosing studies. Such protocol controls overcome some of the known deficits in the method to ensure reproducibility of the procedure.

### 4.4. Insight into Inhaled Dose Efficacy in Rat Hyperoxia BPD Model

Quantitative DD enables a better assessment of the potency of inhaled vitamin A in preventing BPD in neonates. In our original report [1], small but detectable improvements in lung histopathology and histomorphometry were obtained in a neonatal rat hyperoxia model of BPD by intramuscular (IM) injection of 5 U/g, scaled allometrically from the usual NICU dose of 5000 U IM (to a typical premature baby of 1 kg body mass). In that report, for aerosol doses, we inferred from prior studies that loading the nebulizer with 25 U/g would yield a sufficient inhaled dose, but without specific proof of the actual amount of lung-delivered dose. It is clear that effectiveness of the nebulized dose was notably more robust than IM dosing, with inhalation dosing resulting in many lung metrics being statistically comparable with healthy controls despite 7 days of continuous exposure to 95% oxygen. As shown in Figure 1, we now calculate our original dosing scheme [1] to have delivered to the lung about 0.10 U total per aerosol dose at PD7, which is in a range of about 0.01–0.02 U/g (with rats gaining body mass over the period of the experiments, PD = 1–7). Even allowing for potential factors that could reduce data precision (as discussed above), the dose reaching the lung from inhalation dosing appears to be around 1% or less of the total IM dose, yet the inhaled dose produces demonstrably better outcomes. The quantitative DD_BAL_ data support our original hypothesis that inhalation dosing, directly targeting the lung, would be more effective than IM dosing in the prevention of BPD.

Quantitation also helps mitigate some of the obvious hurdles for translation to humans. In particular, our data confirm scaling up the inhalation dosing method from rat pups to lambs, while also validating the quantitation method and reproducibility across two species. Additionally, we have now quantitated the inhaled dose which shows benefit in our hyperoxic rat model [1]. Because the DD_BAL_ appears to be considerably lower than IM dosing administered on a U/g or kg basis, and that IM dose is well known to be generally safe and well tolerated in preterm neonates aside from concerns of intramuscular injection (e.g., see [23]), we can conceivably extrapolate a decreased risk of toxicity of the inhaled route in aerosol toxicology studies and ultimately to humans.

## 5. Conclusions

The use cases presented illustrate potential common issues encountered during aerosol drug development, and how the quantitation of lung-delivered dosing can be especially useful throughout such programs. Having such data allowed us to advance our development while also sparing the use of animals as well as minimizing the time and cost associated with performing the studies and analysis of the data. Although the method was devised and adapted for our specific project, the method itself should find similar utility for other programs that focus on aerosol drug delivery.

## Figures and Tables

**Figure A1:**
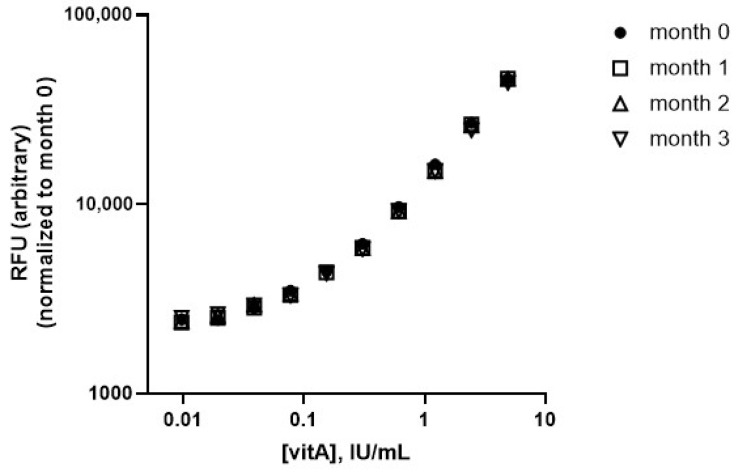
Standard curves from experiments performed a number of months apart (see legend), all using the same batch of the to-be-nebulized fluorescence-bearing formulation. Plate reader configuration was the same in all cases and RFU values are shown normalized to the month 0 data set to facilitate comparison.

**Figure A2:**
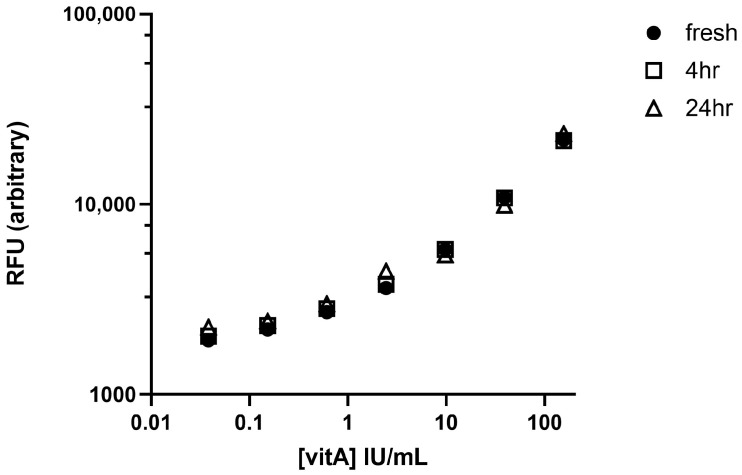
Repeated readings of a prepared standard curve that was allowed to dwell in microtiter plate wells, covered, in the dark, and at room temperature. No attempt was made to precisely control temperature during storage or reading, allowing evaluation of ‘room temperature’ dwell during the overall operation of a typical experiment.

## Data Availability

Data are available on request from the authors.

## Abbreviations

The following abbreviations are used in this manuscript:

BAL	Bronchioalveolar lavage
BALF	Bronchioalveolar lavage fluid
BPD	Bronchopulmonary dysplasia
bw	Body weight
DCM	Dichloromethane
DD	Delivered dose
DD_BAL_	Delivered dose measured in BALF
DD_N_	Delivered dose at the nose
NOAE	Nose-only aerosol exposure
PD	Postnatal day (age)
RFUs	Relative fluorescence units
RMV	Respiratory minute volume
U	Units of vitamin A activity
WBAE	Whole-body aerosol exposure

## Appendix A

### Appendix A.1. Fluorescence Characteristics

The excitation (λ_ex_) and emission (λ_em_) maximum wavelengths of the fluorescence measured in the current work can be compared to the reported characterization of the fluorophore [8], which included the quantum yield (Table A1).

**Table A1:** Fluorescence parameters from our study (top line) compared to wavelengths and quantum yield as reported in [8] (noting that LightOx 23 was also referred to as DC461 in their studies).

Preparation	λ_ex_, Max (nm)	λ_em_, Max (nm)	Quantum Yield (ϕ, %)
Formulation of current report	356	495	n.d.
LightOx 23 in DMSO	362	484	95.4
LightOx 23 in ethanol	327	436	18.65
LightOx 23 in dichloromethane (DCM)	366	491	51.52
LightOx 23 in toluene	360	422	71.06

The fluorescence wavelengths in our micellar system correlate most closely to the fluorescence in DMSO and DCM solvents, suggesting that the microenvironment of the fluorophore in our system matches the properties of DMSO and DCM. It is also reasonable to expect that the quantum yields would match; suggesting our system probably has quantum yields of at least 50%.

The match of wavelengths to DMSO and DCM also provides key information about the microenvironment of the fluorophore in our system, specifically that the fluorescence signal comes from fluorophore located inside the primarily hydrophobic core of the micelles. This is consistent with the fact that the fluorophore and vitamin A palmitate itself are essentially insoluble in water. Our formulation is such that the fluorophore is located inside micelles, resulting in a persistently water-miscible state, and those micellar structures appear to persist throughout the experiments, including the relatively brief time in vivo.

### Appendix A.2. Characterization of Reliability and Stability of the Fluorescence-Bearing Formulation

The stability of the specially prepared fluorescence-bearing formulation can be demonstrated by overlaying standard curves from different experiments (Figure A1) performed over a range of months. Because fluorescence signal is always *relative* (relative fluorescence units, RFUs), we have facilitated comparison here by normalizing to the chronologically earliest standard curve, and then overlayed the standard curve dilution series prepared for experiments 1–3 months later.

This comparison suggests that the fluorescence-bearing formulation is sufficiently stable during refrigerated storage to provide reliable data over at least several months. To ensure accuracy, we always prepared and read the standard curve every day of the animal experiments. It is a simple and trivial exercise to prepare the same dilution series for the standard curve each day, a pragmatic effort that minimizes unknown or unexpected day-to-day variations.

In a separate experiment, a standard curve was prepared in a microtiter plate and stored covered at room temperature for 24 h. Repeated measurements reveal good stability over 24 h (Figure A2).

We did not similarly evaluate the on-plate stability of BALF samples, but, as we described, we focused on reproducibly short dwell times (about 10 min or less) after the collection and preparation of the BALF, which in addition to speedy time to data also minimizes the chance of unknown degradation effects (e.g., onset of biochemical reactions, photodegradation, etc.). We emphasize, extrapolating from Appendix A.1 above, that intact micellar structures shield the fluorophore from chemical damage, at least in the short term, with the micelle forming a durable barrier impeding chemical/biochemical intrusion that could alter the fluorophore molecules contained in the micelle core.
